# Colon Bioaccessibility and Antioxidant Activity of White, Green and Black Tea Polyphenols Extract after In Vitro Simulated Gastrointestinal Digestion

**DOI:** 10.3390/nu10111711

**Published:** 2018-11-08

**Authors:** Giuseppe Annunziata, Maria Maisto, Connie Schisano, Roberto Ciampaglia, Patricia Daliu, Viviana Narciso, Gian Carlo Tenore, Ettore Novellino

**Affiliations:** Department of Pharmacy, University of Naples “Federico II”, Via Domenico Montesano 49, 80131 Naples, Italy; maria.maisto@unina.it (M.M.); connie.schisano@unina.it (C.S.); roberto.ciampaglia@unina.it (R.C.); patricia.daliu@unina.it (P.D.); viviana.narciso@gmail.com (V.N.); giancarlo.tenore@unina.it (G.C.T.); ettore.novellino@unina.it (E.N.)

**Keywords:** tea, polyphenols, bioaccessibility, nutraceutical, microbiota

## Abstract

The beneficial effects of the tea beverage are well-known and mainly attributed to polyphenols which, however, have poor bioaccessibility and bioavailability. The purpose of the present study was the evaluation of colon bioaccessibility and antioxidant activity of tea polyphenolic extract. An 80% methanolic extract (*v*/*v*) of tea polyphenols was obtained from green (GT), white (WT) and black tea (BT). Simulated gastrointestinal (GI) digestion was performed on acid-resistant capsules containing tea polyphenolic extract. The main tea polyphenols were monitored by HPLC-diode-array detector (DAD) method; in addition, Total Phenol Content (TPC) and antioxidant activity were evaluated. After GI digestion, the bioaccessibility in the colon stage was significantly increased compared to the duodenal stage for both tea polyphenols and TPC. Similarly, the antioxidant activity in the colon stage was significantly higher than that in the duodenal stage. Reasonably, these results could be attributable in vivo to the activity of gut microbiota, which is able to metabolize these compounds, generating metabolites with a greater antioxidant activity. Our results may guide the comprehension of the colon digestion of polyphenols, suggesting that, although poorly absorbed in the duodenum, they can exert their antioxidant and anti-inflammatory activities in the lower gut, resulting in a novel strategy for the management of gut-related inflammatory diseases.

## 1. Introduction

Tea is historically recognized as the typical beverage consumed in the oriental tradition, used for more than 5000 years in diet and folk medicine, especially in Asian countries [1]. However, its consumption has increased all over the world, becoming one of the most popular beverages [2]. This spreading is mainly due to the widely accepted beneficial effects of tea on human health, which have been attributed to polyphenols [3], the largest group of phytochemical compounds which includes about 8000 different structures [4]. These compounds are largely contained in several plant-based foods, such as fruits, nuts, tea, coffee and cocoa [5,6], suggesting the pivotal role of their consumption in prevention and management of several diseases, including type 2 diabetes mellitus (T2DM) [6] and cardiovascular disease (CVD) [7]. Most of the main beneficial effects of the Mediterranean Diet, which is recognized as the best health-promoting dietary style, indeed, are attributed to the elevated amount of polyphenols present in its main food constituents [6,7,8,9,10]. Evidence, indeed, suggests that polyphenols, in addition to their well-known antioxidant activity, exert a number of other beneficial effects on human health contributing to preventing and/or managing several pathological conditions, including neurodegenerative diseases, inflammation, cancer, CVD, T2DM and obesity, as recently reviewed by Cory et al. [11]. Among polyphenols, catechins are the most representative in tea (more than 30% of leaf dried weight) [12]. After their synthesis, catechins undergo several esterification reactions with gallic acid, resulting in a number of other bioactive compounds, including (−)-catechin-3-gallate (CG), (−)-epicatechin-3-gallate (ECG), (−)-epigallocatechin (EGC), (−)-epigallocatechin-3-gallate (EGCG), and (−)-gallocatechin-3-gallate (GCG). The EGCG is the most abundant polyphenol in green, white and black tea; ECG and EGC levels are high in white tea, where gallic acid, caffeine and theobromine are also present [13].

Although a number of studies have reported several beneficial effects of tea, it is important to consider that gastrointestinal (GI) digestion is a complex physiological process, which strongly affects structure and activity of diet-derived bioactive compounds, resulting in decreased bioaccessibility and bioavailability. Tenore et al. [14] evaluated in vitro bioaccessibility and bioavailability of polyphenols in black, white and green tea infusions (0.5 g of tea in 20 mL of hot water, 90 °C). Bioaccessibility was investigated using a simulated GI digestion protocol; bioavailability was assessed by a monolayer of Caco-2 human colon carcinoma cell line, as intestinal epithelium experimental model. Results showed a very low intestinal bioaccessibility (about 8%) and bioavailability (2–15% of the intestinal content). The low bioaccessibility is mainly ascribed to the neutral intestinal pH, which causes epimerization and auto-oxidation of catechins. Furthermore, the low catechin transepithelial permeation is probably due to polyphenol instability at neutral pH values and/or the presence of efflux transporters on the apical membrane of intestinal cells [14]. Similar results were obtained by Peters et al. [15] who highlighted that both duodenal bioaccessibility and bioavailability of catechins from green tea were reduced compared to non-digested samples. Interestingly, the same authors demonstrated that these two parameters were enhanced using a formulation of green tea extract with sucrose and ascorbic acid, alone or in combination [15].

Jilani et al. [16] also demonstrated that in vitro GI digestion reduces intestinal bioaccessibility of polyphenols from green and black tea infusions; however, total antioxidant capacity was reduced only in green tea samples, while increased in black tea samples. Additionally, biosorption with *S. cerevisiae* has been proposed as a useful approach to increase polyphenol bioaccessibility. In general, yeast fermentation enhanced both bioaccessibility and antioxidant capacity of tea polyphenols. Specifically, fermented infusions exhibited a lower bioaccessibility than not-fermented; however, in the suspension of *S. cerevisiae* (the pellet obtained after centrifugation of fermented samples) a certain amount of polyphenols was detected. Interestingly, both bioaccessibility and antioxidant capacity of polyphenols in the yeast suspension significantly increased after in vitro GI digestion, suggesting that yeast acted as a good strategy for extracting polyphenols and as delivery system protecting phytochemicals during the GI digestion. This is mainly due to the ability of polyphenols to bind wall components of yeast cells forming complexes with affinities depending on several factors, including chemical structure of polyphenols, protein or polysaccharides concentrations, temperature and pH. According to the authors, the affinity between polyphenols and wall components of yeast cells was higher in black tea samples; this is due to a higher specificity toward high molecular weight polyphenols, such as thearubigins and theaflavins, which also present a high affinity for milk proteins [17,18]. This suggest that food components may also affect bioaccessibility of polyphenols. The formation of complexes between polyphenols and food components may represent a delivery system that protects bioactive compounds from the activity of GI digestion; in turn, changes in pH (in particular, the middle-alkaline pH), variating the affinity of polyphenols, may increase their bioaccessibility.

On the contrary, Coe et al. [19] demonstrated that bioaccessibility of polyphenols from green, white and black tea infusions increased both in gastric and duodenal stages after in vitro GI digestion.

Overall, these data provide information about the metabolic fate of diet-derived bioactive compounds and suggest that, although diet is the main source of bioactive substances, the single or sporadic consumption of foods rich in these compounds is not sufficient to obtain the claimed beneficial effects. The use of nutraceutical products, thus, might represent the best approach to take benefit from their properties.

However, taking into account the physiology of the GI system, a further interpretation of tea polyphenol metabolic fate should be proposed. The prolonged permanence of polyphenols in the intestinal lumen leads to the assumption that these compounds may exert, in situ, their beneficial effects, including the actions on glucose and lipid metabolism [12,13]. Moreover, the non-absorbed polyphenols reach the lower intestine where, before being excreted, they might also exert their antioxidant activity. Interestingly, evidence showed that non-absorbed polyphenols could be metabolized by the microbiota in the colon, resulting in the production of several metabolites, which have higher antioxidant activity [20,21,22].

A limited number of studies have investigated the metabolic fate of tea polyphenols in the large intestine. The purpose of the present study is to investigate the bioaccessibility and antioxidant activity of tea polyphenols in an experimental model of large intestine. Bioaccessibility and antioxidant activity were evaluated using a nutraceutical formulation based on acid-resistant capsules containing 80% methanolic extract (*v*/*v*) of green (GT), white (WT) and black tea (BT). After in vitro simulated GI digestion, significant increases in tea polyphenols and antioxidant activity were observed in the colon stage, as compared to the duodenal stage, suggesting a possible role of gut microbiota in metabolising these compounds in vivo.

## 2. Materials and Methods

### 2.1. Reagents

All chemicals and reagents used were either analytical or HPLC-grade reagents. The water was treated in a Milli-Q water purification system (Millipore, Bedford, MA, USA) before use. Chemicals and reagents used to simulate the gastrointestinal digestion: potassium chloride (KCl), potassium thiocyanate (KSCN), monosodium phosphate (NaH_2_PO_4_), sodium sulphate (Na_2_SO_4_), sodium chloride (NaCl), sodium bicarbonate (NaHCO_3_), hydrochloric acid (HCl) and also the enzymes pepsin (≥250 U/mg solid) from porcine gastric mucosa, pancreatin (4 × USP) from porcine pancreas, protease from Streptomyces griseus, called also Pronase E (≥3.5 U/mg solid), and Viscozyme L were purchased from Sigma-Aldrich (Milan, Italy).

### 2.2. Tea Polyphenolic Extraction

Three variety of tea samples (*C. sinensis*) were purchased in a local market. These were green, white and black tea. All samples were obtained from the same tea cultivar Chun Mee 41022 (Vicony Teas Company, Huangshan, China). For the preparation of the tea polyphenolic extract, 75 mL of 80% methanol was added to 15 g of each dry tea samples, homogenized for 1 min by ultra-turrax (T25-digital, IKA, Staufen im Breisgau, Germania), shaken on orbital shaker (Sko-DXL, Argolab, Carpy, Italy) at 300 rpm for 10 min; the samples were placed in ultrasonic bath for other 10 min and then centrifuged at 6000 rpm for 10 min. The supernatants were collected and stored in the darkness, at 4 °C. The pellets obtained, were re-extracted with other 35 mL of the same mixture, following the procedure previously described. Finally, the extracts were filtered under vacuum, the methanol fraction was eliminated, and the water fraction was lyophilized. The powders obtained were used for the capsules’ formulation. In particular, capsules contained 1000 mg GT, WT or BT polyphenolic extract. The capsules used were acid-resistant (hydroxypropyl cellulose E464, gellan gum E418, hioxide titanium E171).

### 2.3. In Vitro Simulated Gastrointestinal Digestion

The in vitro digestion experiments were performed according to the procedure described by Raiola et al. (2012) [23] and by Tenore et al. (2013) [24], with few modifications. For GI digestion, a capsule was mixed with 6 mL of artificial saliva composed of KCl (89.6 g/L), KSCN (20 g/L), NaH_2_PO_4_ (88.8 g/L), Na_2_SO_4_ (57.0 g/L), NaCl (175.3 g/L), NaHCO_3_ (84.7 g/L), urea (25.0 g/L) and 290 mg of α-amylase. The pH of the solution was adjusted to 6.8 with HCl 0.1 N. The mixture was introduced in a plastic bag containing 40 mL of water and homogenized in a Stomacher 80 Microbiomaster (Seward, Worthing, UK) for 3 min. Immediately, 0.5 g of pepsin (14,800 U) dissolved in HCl 0.1 N was added, the pH was adjusted to 2.0 with HCl 6 N, and the solution was incubated at 37 °C in a Polymax 1040 orbital shaker (250 rpm) (Heidolph, Schwabach, Germany) for 2 h. Then the pH was increased to 6.5 with NaHCO_3_ 0.5 N and 5 mL of a mixture of pancreatin (8.0 mg/mL) and bile salts (50.0 mg/ mL) (1:1; *v*/*v*), dissolved in 20 mL of water, was added and incubated at 37 °C in an orbital shaker (250 rpm) for 2 h. Finally, the mixture was centrifuged at 6000 rpm and the remaining pellets were treated first with 5 mL of 1 mg/mL Pronase E solution (pH 8 for 1 h), and then, with 150 μL of Viscozyme L (pH 4 for 16 h), in order to simulate the colon digestion process, as previously described by Papillo et al. (2014) [25]. Each of the supernatants collected during the different digestion phases simulated were lyophilized, and then dissolved in methanol for the analysis.

### 2.4. Total Phenol Content (TPC)

Total phenol content (TPC) was determined through Folin-Ciocalteau’s method, using gallic acid as standard (Sigma-Aldrich, St. Louis, MO, USA). In brief, 0.1 mL of samples (properly diluted with water in order to obtain an absorbance value within the linear range of the spectrophotometer) underwent an addition of: 0.5 mL of Folin-Ciocalteau’s (Sigma-Aldrich, St. Louis, MO, USA) reagent and 0.2 mL of an aqueous solution of Na_2_CO_3_ (20%; *w*/*v* %), bringing the final volume to 10 mL with water. After mixing, the samples were kept in the dark for 90 min. After the reaction period, the absorbance was measured at 760 nm. Each sample was analyzed in triplicate and the concentration of total polyphenols was calculated in terms of gallic acid equivalents (GAE) [26].

### 2.5. HPLC-DAD Analysis of Tea Polyphenols

The main tea polyphenols were assessed by HPLC/diode-array detector (DAD) analysis, performed using a HPLC system Jasco Extrema LC-4000 system (Jasco Inc., Easton, MD, USA) fitted with an auto sampler, a binary solvent pump, and a diode-array detector (DAD). The separation and quantification were achieved using Synergy Polar-RP C18 column (150 × 4.6 mm I.D., 4 µm particle size, Phenomenex, Torrance, CA, USA) preceded by a Polar RP security guard cartridge. The column temperature was set at 40 °C. The PDA acquisition wavelength was set in the range of 200–400 nm. The mobile phase consisted of water-acetic acid, (97:3 *v*/*v*) (A) and methanol (B). Injection volume was 20 µL and flow rate was kept at 1 mL/min. The gradient program was: 0–1 min %(A), followed by a linear increase of solvent B to 63% in 27 min; then the phase composition was brought back to the initial conditions in 2 min [27]. Calibration curves were obtained at detection wavelength of 280 nm for all catechins using a series of standard dilutions in MeOH, over the concentration range of 0.20–80.0 mg/L.

### 2.6. Antioxidant Activity

#### 2.6.1. DPPH Assay

The antioxidant activity of tea samples was measured with respect to the radical scavenging ability of the antioxidants present in the sample using the stable radical 2,2-diphenyl-1-picrylhydrazyl (DPPH) (Sigma-Aldrich St. Louis, MO, USA). The analysis was performed by adding 100 μL of each sample to 1000 μL of a methanol solution of DPPH (153 mmol L^−1^). The decrease in absorbance was determined with a UV-visible spectrophotometer (Beckman, Los Angeles, CA, USA). The absorbance of DPPH radical without antioxidant, i.e., the control, was measured as basis. All determinations were in triplicate. Inhibition was calculated according to the formula:

[(Ai − Af)/Ac] × 100,(1)

where Ai is absorbance of sample at t = 0, Af is the absorbance after 6min, and Ac is the absorbance of the control at time zero [28]. Trolox was used as standard antioxidant. Results were expressed in mmol Trolox Equivalent (TE).

#### 2.6.2. ABTS Assay

The ABTS assay was performed according to the method described by Rufino et al. (2010) [26] with slight modifications. ABTS solution was prepared [2,20 -azinobis(3-ethylbenzotiazoline-6- sulfonate)] by mixing 5 mL of ABTS 7.0 mM solution and 88 μL of potassium persulfate 2.45 mM solution, which was left to react for 12 h, at 5 °C in the dark. Then, ethanol water was added to the solution until an absorbance value of 0.700 (0.05) at 754 nm (Beckman, Los Angeles, CA, USA). The determination of sample absorbance was accomplished at room temperature and after 6 min of reaction. All determinations were in triplicate. Inhibition was calculated according to the formula:

[(Ai − Af)/Ac] × 100,(2)

where Ai is absorbance of sample at t = 0, Af is the absorbance after 6 min, and Ac is the absorbance of the control at time zero [29]. Trolox was used as standard antioxidant. Results were expressed in mmol Trolox Equivalent (TE).

### 2.7. Statistics

Unless otherwise stated, all the experimental results were expressed as mean ± standard deviation (SD) of three determinations. Statistical analysis of data was performed by the Student’s *t* test or two-way ANOVA (SPSS 13.0) followed by the Tukey-Kramer multiple comparison test to evaluate significant differences between a pair of means. P values less than 0.05 were regarded as significant. The degree of linear relationship between two variables was measured using the Pearson product moment correlation coefficient (R). Correlation coefficients (R) were calculated by using Microsoft Office Excel application.

## 3. Results

### 3.1. In Vitro Bioaccessibility of Tea Polyphenols

Tea polyphenol bioaccessibility was evaluated by using a simulated GI digestion. The use of acid-resistant capsule allowed us to avoid the effects of gastric conditions on the bioactive compounds. For each sample, the gastric bioaccessibility was 0% (Table 1). Equally, the oral bioaccessibility was also 0% (Table 1).

In order to obtain an overview of the bioaccessibility of the tea polyphenols in the various stages of the GI digestion, we firstly evaluated the TPC by Folin-Ciocalteu assay. Table 1 shows the mean values (mg GAE/g) of TPC for GT, WT and BT in each stage of the in vitro GI digestion. In the duodenal stage, TPC was significantly lower than in the not digested samples (*p* < 0.0001 for all samples). On the contrary, in the colon stage TPC significantly increased compared to the duodenal stage (*p* < 0.001, 0.0005 and 0.0001 for GT, WT and BT, respectively). In particular, despite the initial TPC measured in not-digested samples, after in vitro GI digestion WT renders the higher colon bioaccessibility (WT > BT > GT).

**Figure 1:**
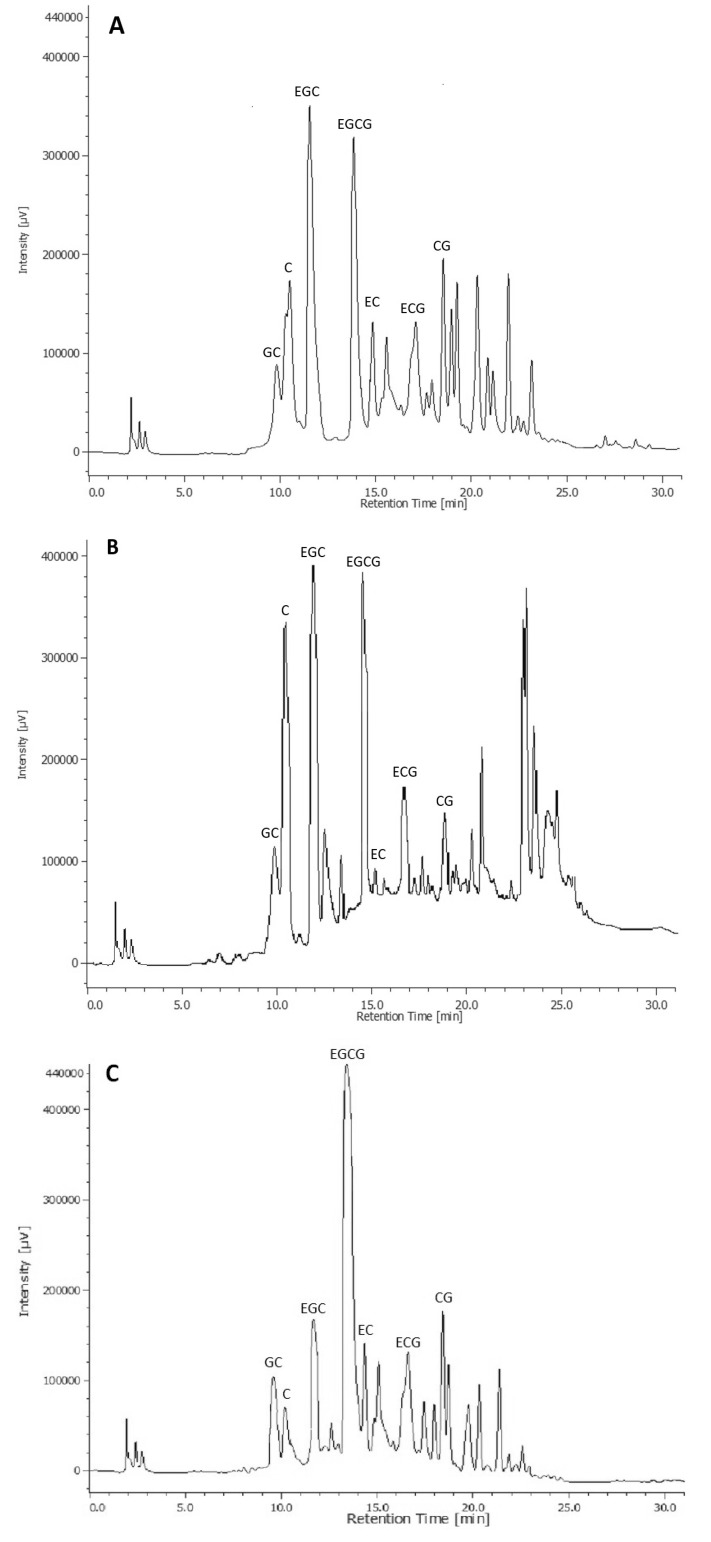
HPLC-diode-array detector (DAD) chromatograms of not digested green tea (GT) (**A**), black tea (BT) (**B**) and white tea (WT) (**C**) with identifying observed catechins. C: (+)-catechin; EC: (−)-epicatechin; ECG: (−)-epicatechingallate; EGC: (−)-epigallocatechin; EGCG: (−)-epigallocatechingallate; GC: (−)-gallocatechin; CG: (−)-catechingallate.

The most representative tea polyphenols were then monitored by HPLC-DAD analysis before and after in vitro GI digestion. HPLC-DAD chromatograms of not digested samples with the identification of the different catechins are reported in Figure 1. Mean values of the main tea polyphenols are reported in Table 2.

As shown in Table 3, interesting data regarding the intestinal bioaccessibility were obtained. In particular, the bioaccessibility in the duodenal stage was significantly reduced compared to not digested samples (*p* < 0.0001 for all samples). On the other hand, the colon bioaccessibility (considered as Pronase E stage + Viscozyme L stage) was significantly higher than duodenal stage (*p* < 0.005 for all samples).

**Figure 2:**
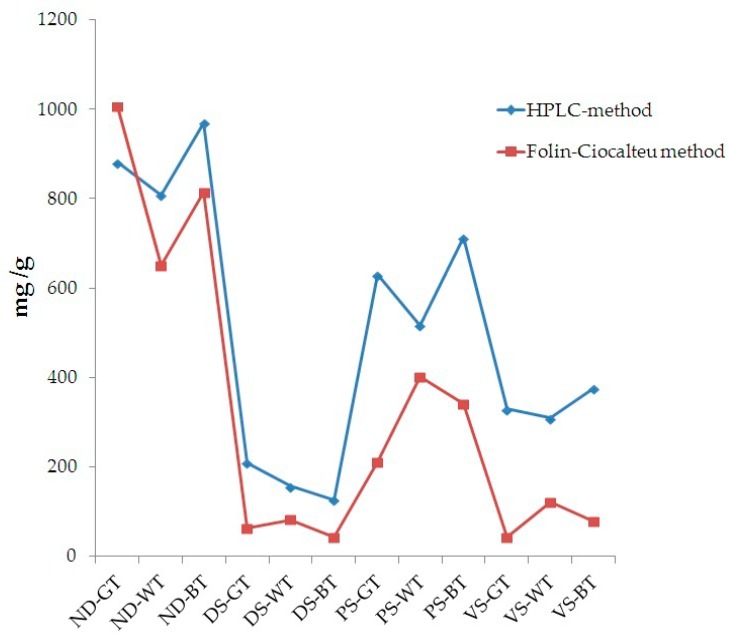
Comparison between the data obtained by the HPLC-DAD method and the spectrophotometric Folin-Ciocalteu method, expressed as mg/g total polyphenols and mg/g gallic acid, respectively.

Data obtained by HPLC-DAD and Folin-Ciocalteu methods were compared (Figure 2). An almost equivalent trend was observed, suggesting that these two methods, although the well-known differences and limitations, provided similar results.

### 3.2. Antioxidant Activity of Tea Polyphenolic Extract after In Vitro Digestion

The antioxidant activity was evaluated by using both DPPH and ABTS assays; results were expressed as mmol of Trolox Equivalent (TE) per g of dried extract. The mean values are reported in Table 4 for each sample in different stages of the in vitro GI digestion.

**Figure 3:**
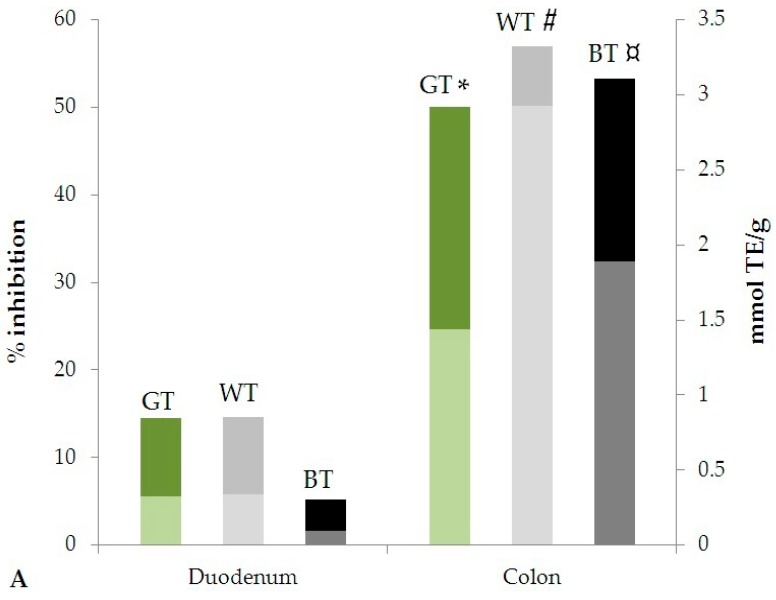

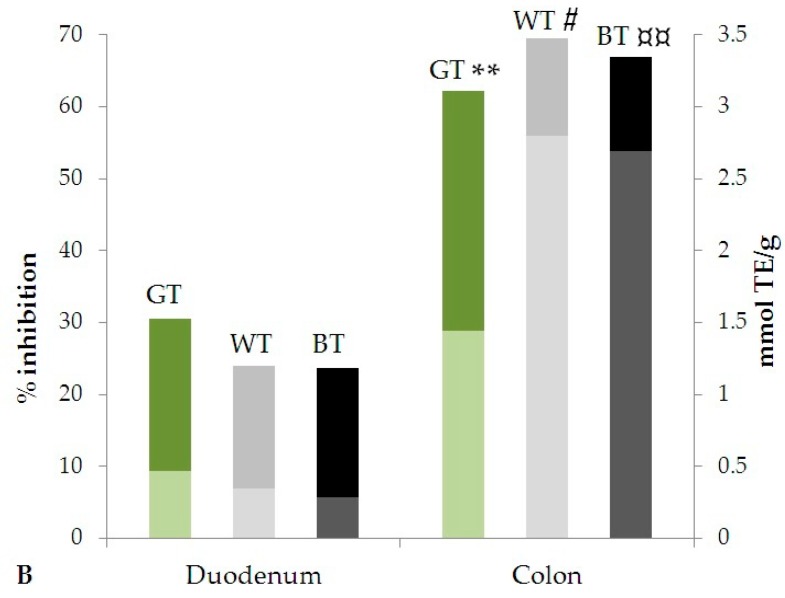
Antioxidant activity evaluated by (**A**) DPPH and (**B**) ABTS methods after simulated in vitro digestion. Statistical significance is calculated by Student’s *t*-test analysis of data expressed in mmol TE/g extract: * *p* < 0.005; ^#^ *p* < 0.01; ^¤^ *p* < 0.0001; ** *p* < 0.05; ^¤¤^ *p* < 0.001, for all Duodenal stage vs. Colon stage (Pronase E + Viscozyme L stages).

For all samples, in both assays, the antioxidant activity in the colon stages was higher than in duodenum. The variation of the antioxidant activity expressed as % inhibition and mmol TE/g in duodenal and colon stages are represented in Figure 3. In particular, a significant increase of the antioxidant activity was observed in the colon stage for both DPPH (*p* < 0.005, 0.01 and 0.0001 for GT, WT and BT, respectively) and ABTS (*p* < 0.05, 0.01 and 0.001 for GT, WT and BT, respectively) assays.

**Figure 4:**
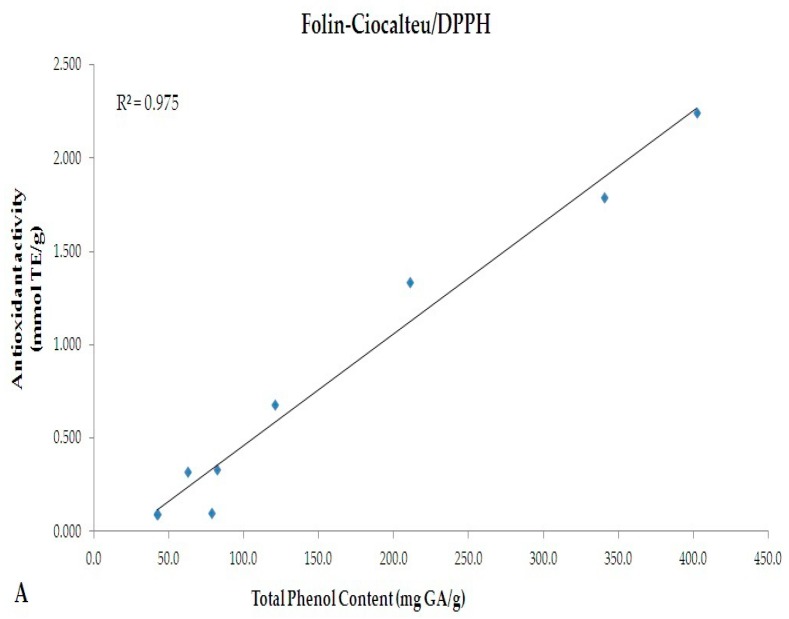

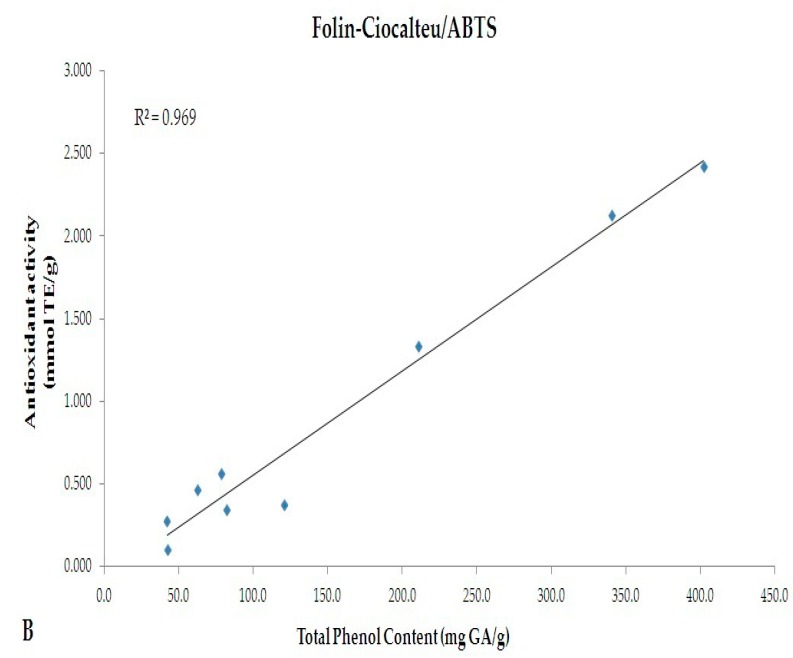
Linear correlation between TPC evaluated by Folin-Ciocalteu (mg GA/g) and antioxidant activity (mmol TE/g) evaluated by (**A**) DPPH and (**B**) ABTS methods.

A linear correlation between the TPC evaluated by Folin-Ciocalteu (mg GAE/g) and antioxidant activity (mmol TE/g) evaluated by DPPH and ABTS methods were performed (Figure 4). A significant correlation was observed between the two spectrophotometric assays (R^2^ = 0.975 and 0.969 for Folin-Ciocalteu vs. DPPH and Folin-Ciocalteu vs. ABTS, respectively).

## 4. Discussion

The present study aimed to evaluate the bioaccessibility and antioxidant activity of tea polyphenols after in vitro GI digestion. The digestion protocol was performed on acid-resistant capsules containing 80% methanolic extract (*v*/*v*) of GT, BT and WT. The use of acid-resistant capsules for the formulation of nutraceutical products represents a useful strategy in order to move bioactive compounds to the intestine, where they can be absorbed or can exert their activities in their active form. Specifically, acid-resistant capsules protect bioactive substances from degradation or alteration of their chemical structure caused by changes in pH or the action of digestive enzymes. A previous study [14] demonstrated that on average 44.4% of native catechin in tea infusions were lost due to gastric digestion and 91.8% after intestinal digestion. Additionally, in the same study, tea polyphenol bioavailability was reported to be very low, suggesting that, overall, GI digestion strongly affects the nutraceutical potential of tea. Thus, taking into account both of these aspects, and the well-established susceptibility of polyphenols to the mild-alkaline conditions, the use of delivery systems is recognized as a novel strategy to increase the amount of bioactive compounds that reach the small intestine, resulting in an increased permeation degree. Data reported in this study might be useful for the formulation of tea polyphenol-based nutraceutical products, which should be formulated under acid-resistant conditions.

As expected, the gastric bioaccessibility was 0%, suggesting that capsules did not decompose during this digestion stage, and polyphenols were not lost Table 1. Similarly, the oral bioaccessibility was 0%, although the oral stage was performed for 3 min. This timing is commonly used for the in vitro digestion of food matrices which undergo chewing, and it seems unrealistic for capsule intake; however, it was performed in order to respect the digestion protocol. Nevertheless, our data suggest that polyphenols are not lost during the oral digestion as well as in vivo when mastication process does not occur after capsule intake, and swallowing is immediate.

The protocol we used for the simulated GI intestinal digestion has been previously performed in our labs and published in various studies [20,21]. In general, it is not too much different from the Infogest method [30] that is recognized as the most eligible method for a comparison of results among different labs using similar and close conditions. In particular, equal timing was kept for each digestive stage (oral stage: 2 min vs. 3 min, Infogest method vs. our method; gastric stage: 2 h; intestinal stage: 2 h). The pH conditions of each stage were similar between the two methods (oral stage 7 vs. 6.8; gastric stage: 3 vs. 2; intestinal stage: 7 vs. 6.5, Infogest method vs. our method). Differences were present among the saline solutions simulating the digestive fluids. The Infogest method uses simulated salivary, gastric and intestinal fluids with standard ions concentrations and volume; on the contrary, we used an artificial saliva that, however, contains several components used in the Infogest method. Similarly, slight differences were among the concentrations of digestive enzymes, although we used the same (α-amylase for the oral stage, pepsin for the gastric stage, pancreatin for the intestinal stage). Nevertheless, these differences may appear as a limitation, it is important to consider that two of the three digestion stages (oral and gastric) were only performed in order to respect the protocol, but variation in the studied matrix or particular results were not expected. As the simulated GI digestion was performed on acid-resistant capsules, oral and gastric digestion did not affect the digested components; thus, these two stages were not relevant. Moreover, during the intestinal stage, the composition of pancreatin we used was similar to that described by the Infogest method. In addition, the main aim of this study was the evaluation of colon digestion that is not contemplate in the Infogest method.

Our main finding in this study is that, after in vitro GI digestion, both bioaccessibility and antioxidant activity of tea polyphenols significantly increased in the colon stage compared to the duodenal stage. Although TPC in not-digested WT was the lowest, our results demonstrate that after simulated GI digestion this kind of tea extract renders the highest duodenal and colonic bioaccessibility, confirming the role of GI digestion in affecting the nutraceutical potential of food-derived extracts. These data suggest that WT extract would benefit the higher polyphenols delivery in both upper and lower intestine.

Bioaccessibility is defined as the amount of polyphenols contained in the water-soluble fraction of each digestion stage, which, in vivo, may be considered as potentially absorbable. As GI digestion is a complex physiological process, in vitro approaches should appear limiting, in particular for the study of digestion in the large intestine, where the activity of microbiota plays a pivotal role. However, the protocol herein used reproductions which were as close as possible to the physiological GI digestion process, as concern chemical, chemical-physical and enzymatic conditions, as well as the average duration of all of the individual stages.

During the simulated GI digestion, a low duodenal bioaccessibility was found for each tea sample (Table 3). This is in agreement with the studies of Tenore et al. [14], Peters et al. [15] and Jilani et al. [16], suggesting that, despite of the fact that polyphenols can be assumed as food or nutraceutical products, their intestinal absorption is low, mainly due to the neutral pH, as mentioned above. This consideration supports the use of delivery systems as strategy to increase the duodenal bioaccessibility.

Physiologically, non-absorbed polyphenols reach the lower gut where they undergo microbial activity [31,32]. This action is due to specific enzymes expressed by bacteria, including carbohydrases which are responsible for both the release of fiber-bound polyphenols and their metabolism [32]. In food matrices polyphenols should exist in the form of glycosides [33]; the presence of a glucose residue strongly reduces both bioaccessibility and bioavailability of phytochemicals. Additionally, some classes of polyphenols, such as catechins, can form oligomers, also called proanthocyanidins or condensed tannins [21]. Simplest catechin (such as monomeric, dimeric and trimeric catechins) are readily absorbed in the small intestine, while catechins with high molecular weight (such as oligomers) have a really poor bioavailability [34], as well as insoluble-bound phenols [35]. In vivo, during the colonic digestion polyphenols may be subjected to hydrolyses by gut microbiota enzymes which are responsible for hydrolytic release of aglycones from *O*-glucoside and carbon-carbon cleavage in the heterocycle and in the aromatic rings [21], resulting in several modifications of the native chemical structures and generation of smaller metabolites with higher antioxidant activity than native compounds [20,21,22]. It is well-established that techniques using fecal inoculum are the most accurate for the study of colonic digestion, mimicking the activity of microbiota. Previous studies proposed further methods based on the use of mix of bacterial enzymes, such as Pronase E and Viscozyme L [25,36]. Pronase E contains a mix of bacterial protease, whereas Viscozyme L is a preparation containing several carbohydrases, including cellulase, arabanase, hemicellulase, β-glucanase and xylanase [25]. The combination of Pronase E and Viscozyme L reproduces the biochemical conditions physiologically occurring in the colon, simulating the action of microbiota on the digested dietary matrix [36]. According to these studies, thus, the increased bioaccessibility and antioxidant activity observed in our experimental model of colon digestion appears not so far from what may occur in vivo for the activity of gut microbiota.

The protocol herein performed to obtain the methanolic extract is not fully selective for polyphenols and, probably, further components from the food matrix may be extracted, including cell-wall polysaccharides, sugar, alcohols or amines which were not investigated in our study. We hypothesized that in our extracts a certain amount of polyphenols were present in the glycoside form, thus, not detectable through the HPLC-DAD method we used. After the in vitro GI digestion, polyphenols might be released from glucose residues by the activities of Pronase and Viscozyme, showing the increase of free polyphenols observed by both Folin-Ciocalteu and HPLC-DAD methods. This is our hypothesis to explain both relative and total increases of polyphenols in the colon stage observed through these two methods, but no further experiments were performed in order to prove it.

Although a comparison between Folin-Ciocalteu and HPLC-DAD methods was performed (Figure 2), indicating the existence of an almost overlapping trend, discrepancies are notable among the values of TPC and tea catechins chromatographically monitored. This is justified by the different approaches used. Folin-Ciocalteu is a simple and highly efficient method which quantifies TPC as gallic acid [37,38]. Several molecules, however, do not react with the Folin-Ciocalteu reagent. This is due to the absence of functional groups, including catechol moieties [39]. Folin-Ciocalteu method, thus, appears useful to approximately determine the TPC, while through HPLC-DAD selected molecules can be monitored. A heterogeneous pattern of phytochemicals may occur in food matrices, and most of them are extractable by the method we used. As indicated by Folin-Ciocalteu method, our samples have a rich phenolic profile; however, the number of catechins we monitored are limited. Data from Folin-Ciocalteu, thus, does not necessarily reflect the levels of catechins chromatographically monitored. Accordingly, during the in vitro GI digestion, polyphenols should be metabolized by the combined activity of Pronase and Viscozyme, resulting in release of smaller molecules (more reactive to Folin reagents) and which are responsible for the increased antioxidant activity. Specifically, variations in the antioxidant activity have been observed during each stages of the in vitro GI digestion. The percentage of decrease in antioxidant activity from not-digested to duodenal stage was almost similar in all samples (DPPH: −91%, −91.5% and −96% for GT, WT and BT, respectively; ABTS: −89%, −91.6% and −90.5% for GT, WT and BT, respectively). After colon digestion, the percentage of decrease followed a different trend (BT < WT < GT), suggesting that, despite the antioxidant activities of not-digested samples, colonic digestion may affect and/or improve the nutraceutical properties of single extract by enhancing its antioxidant capacity (% of decrease from not-digested to colon stage - DPPH: −60.6%, −26.1% and −18.5% for GT, WT and BT, respectively; ABTS: −66.2%, −31.5% and −9.3% for GT, WT and BT, respectively). As mentioned above, the digestion performed in our experimental model of colon would cause metabolism of native polyphenols contained in the extracts and release of both smaller molecules with higher antioxidant activity and polyphenols from components of the food matrix (i.e., cell wall polysaccharides), resulting in variations in antioxidant activities during the GI digestion. Interestingly, data obtained from both DPPH and ABTS tests correlate well with TPC values, as shown in Figure 4 (R^2^ = 0.975 and 0.969, respectively). After the colon digestion, the highest percentage of decrease in TPC was observed in GT (−74.8%); this is perfectly in line with data regarding the colon antioxidant activity.

Interestingly, beside the potential role of gut microbiota in metabolism of phytochemicals, a ‘two-way’ relationship has been previously described between microbiota and polyphenols [40,41]. Evidences reported that polyphenols are able to modulate the gut microbiota [40,41,42,43], acting as bactericidal and bacteriostatic agents [44]. This is mainly due to the ability of polyphenols to bind bacterial membrane proteins, inhibit the glucose inward transport and complex free iron [44,45]. These effects of polyphenols on gut microbiota, however, seem to be strain-specific. Van Duynhoven et al. [44] described a ‘*bifidogenic effect*’ of black tea and its extract, whereas a ‘*prebiotic-like effect’* of polyphenols has been recently reported, showing the ability of polyphenols to favour the growth of specific bacteria, mainly beneficial strains, and reduce the incidence of pathogens [46]. Recent studies reported that GT polyphenols efficiently modulate gut microbiota composition [47,48]; in particular, a reduction of the *Bacteroidetes* to *Firmicutes* ratios was observed in mice fed with high-fat-diet after administration of GT polyphenols [47], suggesting that these phytochemicals may play a pivotal role in managing metabolic diseases through a different mechanism of action from those previously established [47,48].

## 5. Conclusions

In summary, our results show that, after in vitro GI digestion, tea polyphenol bioaccessibility and antioxidant activity are higher in the colon than in the duodenum, suggesting that, in vivo, the gut microbiota might be able to metabolize dietary polyphenols, resulting in an increase of their beneficial effects in the large intestine. This potential effect appears relevant considering that the large intestine is a physiological site of oxidative stress and, in certain instances, inflammation. The use of nutraceutical formulations, thus, represents a novel and useful strategy in order to vehicle a high amount of bioactive compounds to the intestine, where they can exert their beneficial effects. However, although we used an experimental model of colon, according to previous published evidence, we are conscious that our result is not sufficient to directly attribute these actions to the gut microbiota; however, they do represent a starting point for further investigations. Further studies, thus, are needed to identify the metabolites generated after microbiota metabolism in colon, and evaluate their actions on human health.

## Figures and Tables

**Table 1:** Total Phenol Content (TPC) evaluated by Folin-Ciocalteu method. Data are expressed as mean value (mg gallic acid equivalents (GAE)/g extract) ± SD of three repetitions.

Sample		TPC (mg/g) ± SD
Tea Variety	Digestion Stage	
Green	Not digested	1005.703 ± 28.784
	Oral stage	n.d.
	Gastric stage	n.d.
	Duodenal stage	62.507 ± 2.254 ^a,^*
	Pronase E stage	210.448 ± 24.479
	Viscozyme L stage	42.180 ± 10.939
	Total colon stage	252.628 ± 35.048 ^b,^**
White	Not digested	650.654 ± 15.848
	Oral stage	n.d.
	Gastric stage	n.d.
	Duodenal stage	82.053 ± 15.294 ^c,^*
	Pronase E stage	402.221 ± 17.794
	Viscozyme L stage	120.760 ± 38.581
	Total colon stage	522.981 ± 55.831 ^d,^***
Black	Not digested	814.600 ± 6.968
	Oral stage	n.d.
	Gastric stage	n.d.
	Duodenal stage	42.111 ± 1.751 ^e,^*
	Pronase E stage	340.196 ± 15.132
	Viscozyme L stage	78.432 ± 6.288
	Total colon stage	418.628 ± 21.375 ^f,^**

Statistical significance is calculated by Student’s *t*-test analysis: * *p* < 0.0001 Not digested vs. Duodenal stage; ** *p* < 0.001 Duodenal stage vs. Colon stage (Pronase E + Viscozyme L stages); *** *p* < 0.0005 Duodenal stage vs. Colon stage (Pronase E + Viscozyme L stages). ^a,b,c,d,e,f^ Mean values with different superscript letters are significantly different by Tukey-Kramer multiple comparison test. n.d.: not detected.

**Table 2:** HPLC-DAD analysis of the main tea polyphenols.

Sample		Main Tea Polyphenols, Mean Values (mg/g) ± SD							
Tea Variety	Digestion Stage	C	EC	EGCG	ECG	EGC	GC	CG	Tot.
Green	Not digested	112.016 ± 1.493	56.361 ± 0.620	213.260 ± 1.337	101.010 ± 1.322	280.430 ± 0.149	33.735 ± 1.294	84.106 ± 0.146	880.924 ± 6.309
	Duodenal stage	26.618 ± 1.617	13.314 ± 0.153	50.769 ± 0.535	24.085 ± 0.521	66.597 ± 0.100	7.980 ± 0.278	20.012 ± 0.018	209.377 ± 3.151
	Pronase E stage	79.894 ± 1.747	39.823 ± 0.399	152.95 ± 0.889	71.910 ± 1.847	199.8 ± 0.163	25.099 ± 1.016	59.987 ± 0.122	629.466 ± 3.885
	Viscozyme L stage	41.979 ± 1.617	20.833 ± 0.417	79.675 ± 0.429	37.894 ± 0.432	105.010 ± 0.287	12.545 ± 0.881	31.527 ± 0.141	329.469 ± 4.158
White	Not digested	88.646 ± 1.456	33.776 ± 0.539	382.790 ± 1.404	96.294 ± 1.849	98.594 ± 0.522	40.711 ± 1.420	67.486 ± 0.396	808.294 ± 6.662
	Duodenal stage	17.085 ± 1.541	6.432 ± 0.229	74.152 ± 0.369	18.539 ± 0.573	19.017 ± 0.185	7.981 ± 0.528	13.095 ± 0.024	156.302 ± 3.430
	Pronase E stage	56.687 ± 1.902	21.283 ± 0.475	254.32 ± 1.585	61.615 ± 1.630	62.890 ± 0.187	25.856 ± 1.682	43.272 ± 0.290	516.925 ± 7.696
	Viscozyme L stage	34.055 ± 1.564	12.654 ± 0.737	146.580 ± 1.559	36.856 ± 1.406	37.443 ± 0.349	15.848 ± 1.563	25.826 ± 0.278	309.259 ± 7.393
Black	Not digested	231.918 ± 2.085	26.266 ± 1.010	267.080 ± 1.254	92.348 ± 1.423	287.53 ± 0.583	45.322 ± 1.382	18.678 ± 0.483	969.143 ± 8.176
	Duodenal stage	30.177 ± 1.300	3.329 ± 0.297	34.749 ± 0.131	12.010 ± 0.120	37.447 ± 0.177	5.898 ± 0.115	2.423 ± 0.014	126.035 ± 2.147
	Pronase E stage	170.548 ± 2.326	19.353 ± 0.920	196.540 ± 1.562	67.882 ± 1.704	211.710 ± 0.503	33.283 ± 1.634	13.720 ± 0.320	713.038 ± 8.966
	Viscozyme L stage	89.895 ± 1.674	10.133 ± 0.796	103.090 ± 1.392	35.787 ± 0.290	111.420 ± 0.280	17.478 ± 0.639	7.164 ± 0.252	374.968 ± 5.274

Polyphenolic content is expressed as mean value (mg/g tea extract) ± SD (*n* = 3). C: (+)-catechin; EC: (−)-epicatechin; ECG: (−)-epicatechingallate; EGC: (−)-epigallocatechin; EGCG: (−)-epigallocatechingallate; GC: (−)-gallocatechin; CG: (−)-catechingallate.

**Table 3:** Intestinal bioaccessibility of tea polyphenols evaluated by HPLC-DAD method after the simulated in vitro digestion.

Sample	Duodenal Bioaccessibility		Colon Bioaccessibility	
	Total Polyphenols (mg/g)	%	Total Polyphenols (mg/g)	%
Green tea	209.377 *	23.77	958.933 **	108.85
White tea	156.302 *	19.33	826.185 **	102.21
Black tea	126.035 *	13.00	1088.007 **	112.26

Statistical significance is calculated by Student’s *t*-test analysis: * *p* < 0.0001 Not digested vs. Duodenal stage; ** *p* < 0.005 Duodenal stage vs. Colon stage (Pronase E + Viscozyme L stages).

**Table 4:** Antioxidant activity of digested samples evaluated by DPPH and ABTS assays. Data are expressed as mean value in mmol TE/g extract ± SD (of three repetitions).

Sample		Antioxidant Activity (mmol TE/g ± SD)	
Tea Variety	Digestion Stage	DPPH Assay	ABTS Assay
Green	Not digested	3.649 ± 0.342	4.269 ± 0.274
	Duodenal stage	0.325 ± 0.013	0.469 ± 0.187
	Pronase E stage	1.339 ± 0.336	1.335 ± 0.403
	Viscozyme L stage	0.098 ± 0.006	0.108 ± 0.046
White	Not digested	3.961 ± 0.453	4.085 ± 0.213
	Duodenal stage	0.338 ± 0.102	0.344 ± 0.140
	Pronase E stage	2.244 ± 0.743	2.421 ± 0.779
	Viscozyme L stage	0.684 ± 0.073	0.375 ± 0.139
Black	Not digested	2.322 ± 0.206	2.971 ± 0.274
	Duodenal stage	0.093 ± 0.014	0.283 ± 0.039
	Pronase E stage	1.793 ± 0.094	2.129 ± 0.302
	Viscozyme L stage	0.100 ± 0.006	0.564 ± 0.115
